# Effects of visual blur on microsaccades during visual exploration

**DOI:** 10.16910/jemr.12.6.10

**Published:** 2019-08-12

**Authors:** Sherry Tang, Peggy Skelly, Jorge Otero-Millan, Jonathan Jacobs, Jordan Murray, Aasef G. Shaikh, Fatema F. Ghasia

**Affiliations:** Case Western Reserve University School of Medicine, USA; Daroff-Dell’Osso Ocular Motility Laboratory, Louis Stokes Cleveland VA Medical Center, USA; Vestibular and Ocular Motor Research Laboratory, Johns Hopkins University, USA; Vision Neurosciences and Ocular Motility Lab, Cole Eye Institute, USA; Cleveland Clinic, USA

**Keywords:** visual acuity, eye movement, saccades, gaze, microsaccades, blur, region of interest, visual fading

## Abstract

Microsaccades shift the image on the fovea and counteract visual fading. They also serve as an optimal sampling strategy while viewing complex visual scenes. Microsaccade production relies on the amount of retinal error or acuity demand of a visual task. The goal of this study was to assess the effects of blur induced by uncorrected refractive error on visual search. Eye movements were recorded in fourteen healthy subjects with uncorrected and corrected refractive error while they performed a) visual fixation b) blankscene viewing c) visual search (spot the difference) tasks. Microsaccades, saccades, correctly identified differences and reaction times were analyzed. The frequency of microsaccades and correctly identified differences were lower in the uncorrected refractive error during visual search. No similar change in microsaccades was seen during blank-scene viewing and gaze holding tasks. These findings suggest that visual blur, hence the precision of an image on the fovea, has an important role in calibrating the amplitude of microsaccades during visual scanning.

## Introduction

The eye movements evoked during visual exploration comprise of fast saccadic eye movements that bring the object of interest onto the fovea alternating with periods of fixation ( 1 , 2 ). The fovea, which processes the highest resolution of visual information, is known to fixate toward areas of interest in a scene. Saccades are larger shifts in gaze that lead to goal-oriented switches in foveal fixation ( 3 ). Fixations are thought to be periods of relative stillness so that attention can be focused on salient features throughout the environment. However, even during fixation, our eyes are not perfectly still - the eyes produce fixational movements: microsaccades, drifts, and tremor ( 2 ). Recent studies have begun to elucidate the role of fixational eye movements, and the impact of pathologies on these parameters ( 4 ). Microssacades are smaller shifts that have been shown to counteract visual fading from neural adaptation ( 5, 6, 7, 8 ). In addition, it has been shown that microsaccades occur at an increased rate during high-acuity visual tasks, precisely adjusting foveal gaze based on ongoing visual needs ( 9 ).

Visual blur can occur due to a variety of conditions that cause an inability of a focused image to reach the fovea. Myopia and astigmatisms result in visual blurring if the refractive error is uncorrected. Studies have shown that blur has an impact on visual fixation, where more fixations occur in the least blurred region of the visual space, and that these areas are fixated upon first ( 1 ).

Decreased saccade amplitudes are associated with increasingly difficult visual tasks ( 10, 11, 12 ), leading to the hypothesis that smaller saccades are a strategy used for exploring visually complex scenes ( 13 ). Also, increased saccade amplitudes has been reported to be negatively correlated with the time required to complete a visual task ( 10 , 14 ). Saccades and microsaccades are now thought to be on a continuum of eye movements and share a common neurophysiologic basis ( 2 , 15 ) with similar properties ( 2 , 16 , 17 ). Microsaccades, in countering fading, are also though to play a role in visual search, where they allow the brain to sample fine objects, sharp edges, and contrast details, much like how saccades allow for sampling of larger areas in a scene ( 8 , 9 , 13 , 18, 19, 20, 21 ). Loss of visual acuity due to amblyopia is associated with impaired fixational eye movements, including increased drift and decreased production of microsaccades ( 22 ). Given the impact of visual blur on visual search and saccades and the continuum of saccades with microsaccades, investigation of the impact of visual blur on microsaccades is warranted. Microsaccades seem to be dependent on precision of an image on the fovea; visual blur due to refractive errors was shown to correlate with an increase in microsaccade amplitudes in a severity dependent fashion ( 23 , 24 ). This suggests that the calibration of microsaccades relies on retinal image precision. In the present study, we further explore the impact of visual blur on microsaccades by quantifying microsaccade parameters during gaze fixation, viewing of a blank scene, and during visual search. We hypothesize that the frequency of microsaccades will increase with increasingly complex visual tasks.

## Methods

### Participants

We recruited 14 subjects with various refractive errors. The subjects were further divided into groups by the severity of refractive errors as mild (up to -3.00, n=7), moderate (-3.00 to up to -6.00, n=3), and severe (-6.00 and up, n=4) (Table 1). 

**Table 1:** Clinical characteristics and demographics of study participants.

			Acuity*		Refractive Correction	
Category	Gender	Age	OD	OS	OD	OS
Mild	M	37	0	0	-2.5	-2.5
Mild	F	14	0	0	-1.50+0.25x180	-1.50+0.25x180
Mild	F	11	0	0	-1.75	-2.5+0.75x54
Mild	F	37	0	0	-1.75+0.50x10	-1.0+0.5x175
Mild	F	27	0	0	-2.25+0.75x95	-1.75+0.75x 87
Mild	F	38	0	0	-2.25+1.0x80	-.2.50+0.50x80
Mild	M	14	0	0	-0.5	-1.5+0.50 x 80
Moderate	F	26	0.30	0.30	-3.25	-3.25
Moderate	M	30	0.54	0.54	-4.25	-4.25
Moderate	M	15	0.45	0.45	-4.0+0.50x100	-4.25+0.75x85
Severe	M	21	0.8	0.8	-6.0+1.0x 90	-5.75+1.0x90
Severe	F	27	1	1	-6.75+1.0x90	-6.75+1.0x90
Severe	F	33	0.95	1	-7.0+0.5x85	-7.75+0.75x95
Severe	M	17	0.95	0.95	-7.25+1.0x90	-7.0+0.5x100

*Visual Acuity for all the participants expressed in logMAR in uncorrected state at a viewing distance of 55 cm.

### Procedure

**Figure 1.**
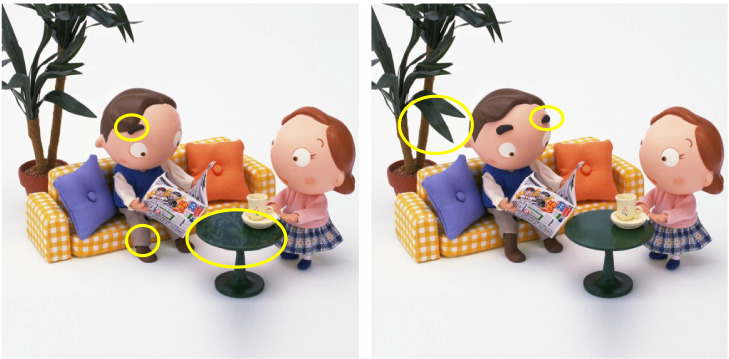
. Example of the visual search task. Yellow circles indicate picture differences. Subjects were asked to identify and click on picture differences. For analysis, each image was divided into 25 areas of interest for a total of 50 per set. Time and location (x, y) of the clicks were recorded. Image from the app Picture Mania Deluxe by Raccoon Digital Technology Co Ltd.

Eye movements were recorded using a high-resolution video-based system (Eyelink) previously described ( 25 , 26 ). The experimental protocols complied with the tenets of the Declaration of Helsinki and were approved by the Cleveland Clinic IRB. Written informed consent was obtained from subjects or parents/legal guardians if subjects were children. Right and left eye calibration was done sequentially under binocular viewing condition. The calibration and validation was done prior to the start of each trial per the manufacturer’s guideline. The participation wore appropriate correction for trials in which data was being collected for corrected refractive error state. All the tasks were done under binocular viewing condition. None of the participants had strabismus. The viewing distance for each task was 55cm. Eye movements with both uncorrected and corrected refractive error were recorded during three tasks: 1) gaze holding: subjects were instructed to fixate their gaze on a circular, red target that subtended a 0.5° visual angle on a white background (luminance 144 cd/m ^2^ ), 2) viewing of a blank scene: subjects were instructed to visually explore a blank scene at 50% gray scale, and 3) visual search task: subjects were asked to identify differences (10 total) between two similar images and to click on areas showing the difference upon identification (Figure 1). Each of the gaze holding, viewing of a blank scene, and visual search tasks lasted for 45 seconds. Subjects were informed of the total number of differences between the images. All images used in the visual search task were displayed on a monitor with a size of 33.7 cm by 27 cm and resolution of 1024x768 pixels. The images presented in the corrected and uncorrected states were randomized. The values of average luminance, minimum and maximum luminance and quantiles of average luminance of each image are in Supplemental Table 1. The time and location of clicks were recorded. A total of 10 sets of images were prepared (5 were tested in corrected refractive error (CRE) and 5 in uncorrected refractive error (URE)). The order of testing in uncorrected and corrected state was randomized and was. A given image was tested across subjects in both corrected and uncorrected states in a randomized fashion. Analyses were performed using Matlab and GraphPad Prism 7 (La Jolla, CA, USA).

### Analysis

Each set of images was divided into a grid (size: 3.3 x 5.5°) containing a total of 50 squares, some of which contained visual differences (areas of interest), such that there were 25 squares per image. We examined behavioral parameters of each subject by analyzing the number of correctly identified picture differences, reaction time to the first identified difference, percent of fixation in areas of interest (those that contain a picture difference) and percent of these fixations that correspond to a click correctly identifying a difference. Paired and unpaired t-tests were used to analyze differences in these parameters between states of corrected and uncorrected refractive errors in each subject as well as in previously mentioned groups (mild, moderate, and severe). Paired t-test were used for gaze holding and blank scene viewing tasks and unpaired t-tests were used for picture differences tasks because subjects were assigned to view different pictures under corrected and uncorrected refractive error conditions. Astigmatism among subjects was all small (≤1 cylinder) and comparable across the groups. Thus, it was thought unlikely to contribute to significant blurriness under binocular uncorrected refractive error conditions.

Oculomotor parameters including microsaccades and saccades were identified from recorded data using the Engbert algorithm ( 23 , [Bibr b27] ). Microsaccades were defined to be any saccade with an amplitude of less than or equal to 1 degree ( 8 , 28 , 29 ). We used composite eye amplitudes of the right eye for analysis. The frequency of microsaccades and saccades (per total task time) were calculated for gaze holding, viewing of blank scene, and visual search tasks. To examine the effect of visual blurring on microsaccade and saccade frequency, frequencies of these in the uncorrected and corrected refractive error states were compared in individual subjects and in severity groups using unpaired t-tests for the picture difference task. The same was done for the gaze holding and blank scene viewing tasks using paired t-tests. Frequencies of microsaccades and saccades corresponding to areas of interest, as well as microsaccades and saccades corresponding to clicks correctly identifying picture differences were also analyzed using unpaired t-tests for individual subjects and in severity groups. To examine the relationship between saccade generation and visual complexity, we used repeated-measures ANOVA to compare the frequencies of microsaccades and saccades of during the three visual tasks.

## Results

Uncorrected refractive error (URE) causes visual blur. We assessed the impact of such visual blur on microsaccade production during the gaze holding task, while viewing a blank scene and while performing the visual search task. We will first review the behavioral and oculomotor performance during the visual search task, followed by eye movement characteristics during viewing of a blank scene and the gaze holding task.

### Visual Search Task

#### Behavioral parameters during visual search task

**Figure 2.**
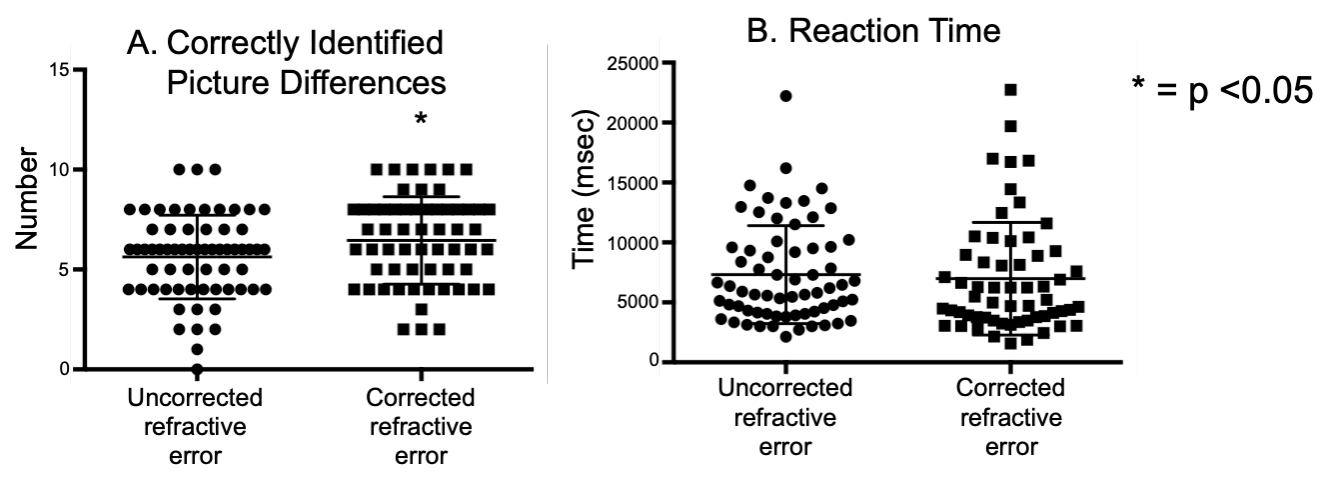
Scatter plot with mean and standard deviation of correctly identified picture differences (A) and reaction time to the first click (B) with uncorrected refractive error (circles) vs. corrected refractive error (squares) condition. Each point represents one trial (total of 5 trials/subject in uncorrected and 5 trials/subject in corrected state). Subjects correctly identified more differences with corrected refractive error. There were no significant differences in the reaction times.

We analyzed the number of correctly identified picture differences in the same subject with and without correction. Overall subjects identified fewer picture differences (Fig 2A) in trials with uncorrected refractive error than those with corrected refractive error (URE: 5.6 ± 2.0, CRE: 6.4 ± 2.1, unpaired t test =0.03). There did not appear to be a difference in reaction times between the uncorrected versus corrected state (URE: 7.32 ± 4.0, CRE: 6.98 ± 4.6, unpaired t test =0.6) (Fig 2B). We analyzed the differences as a function of severity of uncorrected refractive errors and found that the severe myopic subjects had greater difficulty in spotting the differences in the uncorrected state (URE: 5.0 ± 2.5, CRE: 6.9 ± 1.9, unpaired t test =0.02). No similar differences were seen in mild and moderate myopic groups.

#### Oculomotor parameters during visual search task

**Figure 3.**
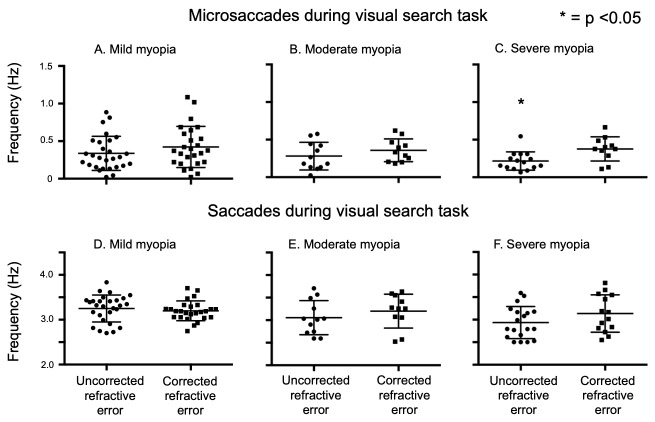
Scatter plot with mean and standard deviation of microsaccade (A-C) and saccadic (D-F) frequencies by the severity of myopia in uncorrected and corrected refractive error conditions during the visual search task. Each point in all the plots represents data obtained during one spot the difference trial. Frequency of microsaccades generated was higher with corrected refractive error in the severe myopia group. There were no significant differences in the frequency of saccades between the three groups.

Frequency: We analyzed the frequency of microsaccades and saccades elicited during the visual search task in the corrected and uncorrected states. Microsaccade frequency in the uncorrected trials was decreased compared to the corrected trials (URE: 0.29 ± 0.19 Hz, CRE: 0.39 ± 0.22 Hz; p=0.01, unpaired t-test). There was no significant decrease in the saccade frequency in the URE trials as compared to CRE trials (URE: 3.1 ± 0.35 Hz, CRE: 3.1 ± 0.31 Hz; p=0.26, unpaired t-test). When the same trials were analyzed by dividing subjects into groups based on the severity of their refractive errors (Fig 3A, B and C), only the severe myopia group showed a statistical difference between the uncorrected and corrected refractive errors [(Mild Myopia: URE = 0.33±0.22, CRE = 0.42±0.27; unpaired t test p=0.2) (Moderate Myopia: URE = 0.28±0.18, CRE: 0.35±0.15: unpaired t test p=0.2) (Severe Myopia URE = 0.21 ± 0.12, CRE: 0.37±0.16; unpaired t test p=0.007)]. No such difference in frequencies of saccades were noted, when dividing the subjects into groups based on the severity of myopia (Fig 3 D, E and F), [(Mild Myopia: URE = 3.2 ±0.31, CRE: 3.1 ± 0.2; unpaired t test p=0.2) (Moderate Myopia: URE = 3.05 ± 0.37, CRE = 3.2 ± 0.37; unpaired t test p=0.2) (Severe Myopia: URE = 2.9 ± 0.35, CRE = 3.13 ±0.41; unpaired t test p=0.14)].

We also assessed the frequency of microsaccades that occurred while the subjects were viewing the image in areas where there were picture differences (regions of interest). We found no statistically significant differences in the frequency of microsaccades produced across different subgroups in the corrected and uncorrected state.

**Figure 4.**
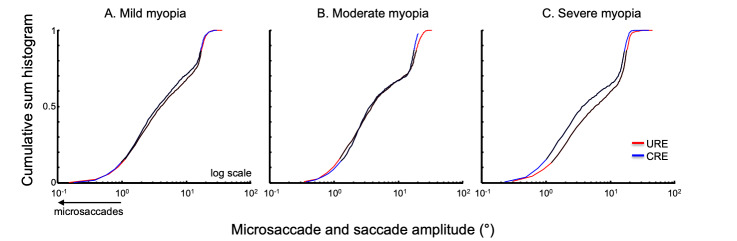
(A-C) microsaccade and saccade amplitudes with uncorrected refractive error (URE) and corrected refractive error (CRE). Microsaccades are those less than 1° in amplitude (arrow). (C) Amplitudes of microsaccades and saccades are larger in subjects with high myopia as indicated by the rightward shift.

Amplitude: It is thought that microsaccades and saccades are an oculomotor continuum. Thus, we analyzed the amplitudes of the microsaccades and saccades collectively for a given trial. Figure 4 summarizes the normalized cumulative sum histogram of the microsaccades and saccades of the right eye elicited during corrected and uncorrected refractive error trials in patients with mild myopia (4A), moderate myopia (4B) and severe myopia (4C). There is a rightward shift of the distribution in the uncorrected state in subjects with severe myopia.

We computed the percentile of the amplitude of the right eye for each subject in corrected and uncorrected state. We then pooled these values per the severity of myopia. We found that the 10th, 25th, 50th and 75th percentiles were different in severe myopia with larger amplitudes in uncorrected state (Table 2). This is consistent with the rightward shift of the cumulative sum histogram in the uncorrected state for severe myopia group. It is noteworthy, that not only the microsaccades but also the saccades especially the mid range ones had greater amplitude in the uncorrected state than corrected refractive error condition.

**Table 2:** Percentile Amplitude of Microsaccade/Saccade elicited in the right eye of subjects in corrected and uncorrected state as a function of severity of myopia during visual search task.

Percentiles Mild Myopia	Corrected	Uncorrected	Paired t test
10^th^	1.02 ± 0.33	1.06 ± 0.31	0.67
25^th^	1.78 ± 0.53	1.79 ± 0.48	0.98
50^th^	4.01 ± 1.27	4.26 ± 1.18	0.50
75^th^	13.17 ± 2.81	14.02 ± 1.89	0.35
90^th^	17.55 ± 1.84	18.03 ± 1.22	0.30
Percentiles Moderate Myopia			
10^th^	1.12 ± 0.09	1.16 ± 0.24	0.90
25^th^	2.02 ± 0.31	1.91 ± 0.49	0.61
50^th^	4.13 ± 1.66	4.47 ± 1.26	0.64
75^th^	14.77 ± 1.61	15.03 ± 3.55	0.86
90^th^	18.03 ± 0.82	19.85 ± 2.05	0.05
Percentiles Severe Myopia			
10^th^	0.92 ± 0.17	1.21 ± 0.27	0.004
25^th^	1.65 ± 0.37	2.21 ± 0.47	0.003
50^th^	4.29 ± 1.91	6.69 ± 2.25	0.0087
75^th^	14.7 ± 1.36	16.03 ± 2.10	0.08
90^th^	17.36 ± 1.15	18.73 ± 1.81	0.04

**Figure 5.**
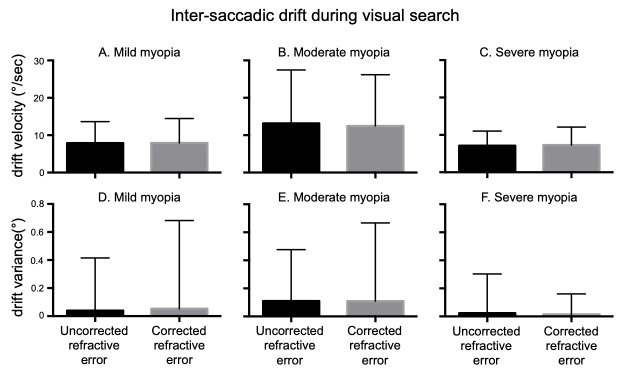
Mean and standard deviation of inter-saccadic drift velocities (A-C) during visual search. There were no significant differences in drift velocities with uncorrected vs corrected refractive error as a function of severity of myopia. Mean and standard deviation of inter-saccadic drift variance (D-F) during visual search task. There were no significant differences in drift variance of eye position in uncorrected vs corrected refractive error as a function of severity of myopia.

Intra-saccadic drift: (Figure 5 A-C) summarizes the mean eye velocities of the right eye elicited during the epochs of inter-saccadic drifts, which were similar in uncorrected and corrected refractive error irrespective of the severity of myopia (Mild Myopia: URE=7.8°/s ± 5.7°/s, CRE= 7.9°/s ± 6.5 /°/s; Moderate Myopia: URE=13.18°/s ± 14.26°/s, CRE= 12.45°/s ± 13.73 °/s; Severe Myopia: URE=7.13°/s ± 3.90°/s, CRE= 7.29°/s ± 4.78 °/s). We also measured the variance of eye positions during inter-saccadic drift as a function of severity of myopia (Figure 5 D-F). There was no difference in drift variance in uncorrected versus corrected state across all three groups (Mild Myopia: URE=0.03° ± 0.37°/s, CRE= 0.05° ± 0.62 /°s; Moderate Myopia: URE=0.1° ± 0.36°, CRE= 0.1° ± 0.55°; Severe Myopia: URE=0.02° ± 0.27°, CRE= 0.01° ± 0.14°).

### Viewing of a blank scene

#### Oculomotor parameters during viewing of a blank scene

Frequency: We assessed the frequency of microsaccades that occurred while viewing a blank scene. We found no statistically significant difference in the frequency of microsaccades produced in the corrected and uncorrected state (URE: 0.17±0.14 Hz, CRE: 0.21±0.16 Hz, paired t test p =0.56). Similarly, there was no difference in the frequency of saccades elicited in the corrected and uncorrected state during viewing of blank scene (URE: 1.2 ± 0.65 Hz, CRE: 1.2 ± 0.83 Hz, paired t test p =0.9).

When divided into groups by myopia severity, microsaccade frequency in uncorrected and corrected trials showed no differences [(Mild Myopia URE: 0.19±0.17, CRE: 0.16±0.12); (Moderate Myopia URE: 0.11±0.11, CRE: 0.08±0.06); (Severe Myopia URE: 0.19±0.16, CRE: 0.36±0.15)]. Similarly, no differences were seen in the frequency of saccades produced in the corrected and uncorrected state across severity of myopia (Mild Myopia URE: 1.18±0.79, CRE: 1.3±0.97); (Moderate Myopia URE: 1.14±0.76, CRE: 0.89 ± 0.75); (Severe Myopia URE: 1.5±0.34, CRE: 1.4±0.80)].

Amplitude: We computed the percentile of the amplitude of the right eye for each subject in corrected and uncorrected state. Unlike in the visual search task, we did not find any difference in the percentile amplitude of the right eye for each subject in the corrected and uncorrected state (Table 3).

**Table 3:** Percentile Amplitude of Microsaccade/Saccade elicited in the right eye of subjects in corrected and uncorrected state during viewing of a blank scene.

Percentiles	Corrected	Uncorrected	Paired t test
10^th^	0.88 ± 0.79	1.10 ± 0.86	0.67
25^th^	1.41 ± 1.04	1.65 ± 1.27	0.98
50^th^	2.60 ± 1.54	2.83 ± 1.82	0.50
75^th^	4.90 ± 2.70	4.89 ± 3.16	0.35
90^th^	7.32 ± 4.36	7.52 ± 4.81	0.74

We compared the 10^th^, 25^th^, 50^th^, 75^th^ and 90^th^ percentile amplitudes for mild, moderate and severe myopia groups and found no difference between corrected and uncorrected state (Mann Whitney test p>0.05 for all comparisons).

### Gaze holding

Frequency: We assessed the frequency of microsaccades that occurred during a simple visual fixation task in the corrected and uncorrected refractive error trials for each subject. We found no statistically significant difference in the frequency of microsaccades produced in the corrected and uncorrected state (URE: 0.47 ± 0.35 Hz, CRE: 0.40 ± 0.30, paired t test p =0.65). However, there was an increase in the frequency of fixational saccades (defined as > 1°) in the uncorrected refractive error trials compared to corrected refractive error trials (URE: 0.46 ± 0.3 Hz, CRE: 0.28 ± 0.21Hz, paired t test p =0.01).

When divided into groups by myopia severity, microsaccade frequency in URE and CRE trials showed no statistically significant differences [(Mild Myopia URE: 0.41±0.12, CRE: 0.47±0.33); (Moderate Myopia URE: 0.48±0.44, CRE: 0.52±0.43); (Severe Myopia URE: 0.52±0.16, CRE: 0.25±0.01)].

Amplitude: We computed the percentile of the amplitude of the right eye for each subject in the corrected and uncorrected state. We found a difference in the 10 ^th^ and 25 ^th^ percentile amplitude of the right eye for each subject with larger amplitudes in the uncorrected state (Table 4).

**Table 4:** Percentile Amplitude of Microsaccade/Fixational saccades elicited in the right eye of subjects in corrected and uncorrected state during simple fixation task.

Percentiles	Corrected	Uncorrected	Paired t test
10^th^	0.25 ± 0.08	0.33 ± 0.10	0.03
25^th^	0.36 ± 0.10	0.41 ± 0.12	0.04
50^th^	0.53 ± 0.18	0.60 ± 0.22	0.31
75^th^	0.77 ± 0.27	0.89 ± 0.52	0.35
90^th^	1.04 ± 0.47	1.19 ± 0.72	0.53

Intra-saccadic drift:Similar to the visual search and blank scene viewing tasks, we found no difference in drift velocity (URE: 5.8°/s ± 1.61°/s and CRE: 6. 3°/s ± 2.9°/s, paired t test: p=0.76) or drift variance (URE: 0.02° ± 0.02° and CRE: 0.02° ± 0.01°, paired t test: p=0.79) in corrected and uncorrected refractive error trials.

Comparison of microsaccade production across all three tasks in corrected and uncorrected state: Microsaccade production was greatest during gaze holding followed by visual search and was lowest during blank scene viewing in corrected refractive error state (Gaze holding: 0.40 ± 0.30 Hz, Visual Search: 0.34±0.19 Hz Blank scene: 0.21±0.16 Hz Friedman test ANOVA: p= 0.04). We found a similar increase in microsaccades during visual fixation in the uncorrected state although it did not reach statistical significance (Gaze holding: 0.47 ± 0.35 Hz, Visual Search: 0.23 ± 0.12 Hz Blank scene: 0.17 ± 0.14,Hz, Friedman test ANOVA: p= 0.07).

## Discussion

We investigated the effects of visual blur on oculomotor performance while exploring a complex visual scene. Subjects generated more microsaccades with corrected refractive errors as compared to uncorrected refractive errors. When the subjects were divided into groups, only those with severe myopia retained a statistically significant difference in microsaccades production. There was no statistically significant difference in microsaccades generated when we only compared areas in the images that contained differences (areas of interest). Furthermore, there were no significant differences in the frequency of saccades generated during trials with corrected and uncorrected refractive error, both overall and when subjects were divided into groups. In addition to frequency, we also observed amplitude differences during the visual search task. In the severe myopia group, both microsaccades and saccades had larger amplitudes with uncorrected refractive errors. Both microsaccades and saccades share a common neurophysiologic circuitry ( 4 , 8 , 17 , 30 ). Thus, a similar change of increased amplitudes for both microsaccades and saccades in the severe myopia group is expected. We found similar intra-saccadic drift velocities and drift variance between trials with corrected and uncorrected refractive errors irrespective of severity of myopia. This suggests that visual blur does not appear to have an impact on the inter-saccadic drift and that the change in microsaccade amplitude is not a result of an increase in the inter-saccadic drift.

We also found that during the visual search task, subjects correctly identified fewer picture differences during uncorrected refractive error trials as compared to corrected refractive error trials. When analyzed by groups, as expected, subjects with severe myopia showed a significant difference, while those with mild and moderate myopia did not. Thus, a decrease in microsaccade frequency in the uncorrected refractive error state in severe myopia was associated with an impaired ability to correctly identify picture differences. This is consistent with the idea that microsaccade generation depends on visual feedback and calibration of microsaccades is dependent on precision of an image on fovea. Thus, decreased visual acuity from visual blur leads to increased amplitude of microsaccades during visual scanning. This is in agreement with previous studies that have found a similar change in microsaccade production during visual search/scanning tasks ( 9 , 10 , 14 , 23 , 26 ). The visual cortex generates the command for microsaccades while the superior colliculus mediates the motor control behind the microsaccades production ( 31, 32, 33 ). Impaired image clarity on the fovea arising from uncorrected refractive error may therefore impact the microsaccade generation and calibration at the level of the visual cortex. Interestingly, there were no differences in the frequencies of saccades generated during the picture difference task during uncorrected refractive error state as compared to corrected refractive error trials. In other words, when the general visual structure of the images are similar, a similar number of saccades may be required to explore the overall scene but spotting smaller differences leads to a different number microsaccades if there is visual blur. This further supports the idea that microsaccades allow for exploration of detailed and complex visual stimuli ( 8 , 18, 19, 20, 21 ). In addition, our results show that free viewing of a blank scene generated the least frequent microsaccades, followed by the visual search task, then by the visual fixation task. There were no differences in the frequency of microsaccades or saccades generated with corrected versus uncorrected refractive error during the blank scene. We did find an increase in the frequency of fixational saccades (> 1°) during straight gaze holding task in agreement with prior study ( 23 ). The properties of visual fixation target are known to affect microsaccades dynamics with viewing of a bigger target eliciting microsaccades with larger amplitude ( 34 ). Thus, an alternate hypothesis is that visual blur of the foveal image due to uncorrected refractive error is equivalent to viewing a larger target resulting in an increase in microsaccade amplitude. However, our study also showed that the frequency of microsaccades increase from free viewing of a blank scene to searching for picture differences to visual fixation, consistent with previous reports that note increasing microsaccade frequency with increasing visual task complexity ( 7 , 9 , 22 , 23 , 35 ).

During tasks such as visual search, both the vision and cognition contribute to the successful identification of targets ( 10 ). Visual impairment is known to impact performance on visual search tasks such as seen in amblyopia. Decreased visual acuity in amblyopic patients leads to greater difficulty completing visual search tasks ( 35 ). In our prior study, we have shown that subjects with more severe amblyopia needed longer to identify visual differences and identified fewer of them correctly. Microsaccade and saccade frequencies were both reduced with increasing severity of amblyopia ( 35 ). Taken together, these further support the idea that saccades and microsaccades are both very important eye movements for visual exploration ( 4 , 8 , 17 , 30 ). Furthermore, it highlights the importance of performing visual search tasks with best-corrected vision as visual blur itself impacts performance on visual search tasks ( 23 , 26 ). In addition to accurate visual input, higher-order processing is also required during completion of visual search tasks ( 36 , 37 ). Higher attention load is associated with an increase in microsaccades ( 27 , 38 , 39 ), pointing to an important role of cognitive processing involved in visual search. In subjects with cognitive impairment due to neurocognitive diseases such as Parkinson’s disease, performance on visual search tasks show an impairment that correlates with disease severity ( 40 ). In addition, patients with Parkinson’s disease and cognitive impairment also showed increased length of fixation on salient visual features when compared with those with Parkinson’s disease with normal cognition ( 40 ). This further supports the idea that fixations bring attention to visually informative features of the scene and that the direction of fixation can indicate foci of attention ( 3 , 41 ). Saccade amplitudes have been shown to be impacted by the presence of cognitive impairment, in that Parkinson’s disease patients with cognitive impairment tended to have smaller saccade amplitudes when compared to those without impairment ( 40 ). This is consistent with command-generating center(s) that direct saccades during visual search ( 10 , 31, 32, 33 ), although it is unclear if these are impaired a cortical level or at the level of oculomotor control in Parkinson’s patients.

There are reports indicating that several characteristics of the visual scene can have an impact on vision during visual exploration ( 2 , 3 ). Features such as luminance, contrast and spatial frequency seem to play a role in where subjects tend to fixate their gaze upon presentation of the visual stimuli ( 3 , 12 , 41 ). In our trials, luminances of the images were normalized, however effects of diminished contrast sensitivity and grating acuity in the uncorrected state were not quantified in these subjects. This may have contributed to our findings of changes in microsaccade production during uncorrected refractive error state and future studies should delve into impact of visual blur on these parameters.

Besides visual stimuli characteristics, eye movements, particularly drift measurements can be affected by the noise with the use of video-trackers. Most sources of noise, such as eyelids/eyelashes covering part of the pupil, poor illumination of the pupil, fuzzy edges of the pupil, etc. would not only increase the noise in the position signal but also the pupil signals. Thus, to delineate the effects of noise, we assessed the changes in the pupil size as an independent measure from the actual eye movements. To assess the effects of corrective lenses on the pupil size, we measured the root mean square of pupil size in corrected and uncorrected states across myopia severity. We found similar RMS values of the pupil across the three groups with and without correction. We also found a small increase in the pupil size with correction across all three groups irrespective of the severity of myopia. However, when we compared the drift velocities, there was no correlation with increasing drift in corrected versus uncorrected state  – thus making it less likely that the change in pupil size is affecting the drift measurements.

To summarize, the results of the current study are consistent with the idea that saccades and microsaccades are a continuum of eye movements. The results also suggest that microsaccades play an important role in visual exploration and their calibration is affected in the presence of visual blur.

## Acknowledgements

This work was supported by grants from Knights Templar Research Foundation (FG), and Fight for Sight Foundation (FG), Blind Children’s Center (FG), RPB Unrestricted grant CCLCM-CWRU, Dystonia Medical Research Foundation clinical fellowship award (AS), Departmental NEI – NRSA T 32 Grant (JM) and Dystonia Coalition Career Development Award.

## Appendix

### Supplemental table 1

**Table 1:** Luminance information on images used during picture difference tasks

				Quantile Average Luminances			
Image	Average Luminance	Minimum Luminance	Maximum Luminance	1 ^ st ^ Quartile	2 ^ nd ^ Quartile	3 ^ rd ^ Quartile	4 ^ th ^ Quartile
1	147.68	17.00	255.00	74.92	129.23	171.10	215.47
2	101.80	1.00	255.00	38.79	72.45	113.17	182.82
3	112.50	6.00	255.00	53.05	87.22	121.91	187.80
4	179.86	4.00	255.00	60.39	178.62	234.40	246.03
5	188.37	3.00	255.00	119.28	181.01	214.25	238.95
6	200.62	3.00	255.00	103.13	206.81	243.98	248.55
7	172.50	17.00	255.00	89.61	166.97	205.12	228.29
8	165.56	31.00	255.00	123.05	153.56	168.19	217.45
9	203.74	25.00	255.00	153.26	201.85	221.65	238.22
10	201.81	3.00	255.00	105.41	209.05	244.06	248.70
